# Targeting Channels and Transporters in Protozoan Parasite Infections

**DOI:** 10.3389/fchem.2018.00088

**Published:** 2018-03-27

**Authors:** Anna Meier, Holger Erler, Eric Beitz

**Affiliations:** Department of Pharmaceutical and Medicinal Chemistry, Christian-Albrechts-University of Kiel, Kiel, Germany

**Keywords:** drug target, transport, infection, resistance, parasite, malaria, protozoa

## Abstract

Infectious diseases caused by pathogenic protozoa are among the most significant causes of death in humans. Therapeutic options are scarce and massively challenged by the emergence of resistant parasite strains. Many of the current anti-parasite drugs target soluble enzymes, generate unspecific oxidative stress, or act by an unresolved mechanism within the parasite. In recent years, collections of drug-like compounds derived from large-scale phenotypic screenings, such as the *malaria* or *pathogen box*, have been made available to researchers free of charge boosting the identification of novel promising targets. Remarkably, several of the compound hits have been found to inhibit membrane proteins at the periphery of the parasites, i.e., channels and transporters for ions and metabolites. In this review, we will focus on the progress made on targeting channels and transporters at different levels and the potential for use against infections with apicomplexan parasites mainly *Plasmodium* spp. (malaria) and *Toxoplasma gondii* (toxoplasmosis), with kinetoplastids *Trypanosoma brucei* (sleeping sickness), *Trypanosoma cruzi* (Chagas disease), and *Leishmania* ssp. (leishmaniasis), and the amoeba *Entamoeba histolytica* (amoebiasis).

## Human-pathogenic protozoa, current treatment, resistance

### Apicomplexa

With more than 200 million new infections per year, malaria-causing *Plasmodium* spp. are the most prominent parasites. The death toll of malaria is still >400,000 per year. About 90% of the cases occur in the WHO African Region and are caused almost exclusively by the species *Plasmodium falciparum*. In the subtropical zones outside of Africa, *Plasmodium vivax* is responsible for up to 64% of the cases. Ongoing efforts to eradicate malaria are hampered by the lack of effective antisera and the spreading of resistant strains against antimalarial treatment (WHO, 2017b). Hence, current research aims at identifying suitable epitopes for future immunization programs, and discovery of new drug targets to establish novel modes of antimalarial drug action.

The current first-line treatment of uncomplicated *P. falciparum* malaria is an oral artemisinin-based combination therapy (WHO, 2015). The mechanisms of how artemisinin and its derivatives, such as artesunate and artemether, attain antimalarial activity are thought to reside in generating oxidative stress by liberating reactive oxygen species from an internal peroxo-moiety, and in affecting a calcium ATPase (SERCA or PfATP6) of the sarcoplasmic-endoplasmic reticulum (Moore et al., 2011). The half-life of the fast-acting artemisinins is very short, typically around 1 h. To maintain this highly efficient therapeutic option in view of an increasing number of mutant parasite strains with varying degrees of resistance to the artemisinins (Jambou et al., 2005), these compounds are combined with drugs that exhibit longer half-lives (WHO, 2001; Kavishe et al., 2017). Patients infected with *P. vivax, Plasmodium ovale, Plasmodium malariae*, or *Plasmodium knowlesi* are equally treated with an artemisinin combination therapy or with chloroquine depending on the sensitivity of the infecting strain. For a number of decades, chloroquine was used for monotherapy until massive resistance occurred. Chloroquine accumulates in the parasite's digestive vacuole and interferes with heme-detoxification during hydrolysis of hemoglobin from the host (Slater, 1993; Thomé et al., 2013; WHO, 2015). For preventing relapse from dormant liver stages after infections with *P. vivax* or *P. ovale*, the use of primaquine is recommended (Fernando et al., 2011; Mikolajczak et al., 2015; Lalève et al., 2016). Complicated infections require rapid administration via intravenous or intramuscular injections of artesunate for at least 24 h followed by artemisinin-based combination therapy (Abiodun et al., 2013; WHO, 2015).

A malaria-related, tick-borne disease, babesiosis, normally occurs in livestock and domestic animals, and only occasionally emerges in humans. Most cases of human babesiosis are caused by *Babesia microti*. First-line treatment is a combination of the ubiquinone analog atovaquone and the antibiotic macrolide azithromycin (Krause et al., 2000).

Another apicomplexan parasite, *Toxoplasma gondii*, causes the food-borne disease toxoplasmosis. It is estimated that 30–50% of the world's population are infected with this parasite. It persists, often life-long, in the host in a dormant, cystic bradyzoite form. Although the infection usually occurs asymptomatic, it can evolve to a life-threating illness in immune-compromised patients. Infections of pregnant women can be transmitted to the fetus giving rise to spontaneous abortion or stillbirth (Flegr et al., 2014). Toxoplasmosis is treated with the dihydrofolate reductase inhibitor pyrimethamine or the antibiotic sulfadiazine. Second-line drugs are azithromycin, clarithromycin, atovaquone, dapsone, and cotrimoxazole. Due to side effects and ineffectiveness against the dormant bradyzoite form novel therapeutics are urgently needed (Petersen and Schmidt, 2003).

### Kinetoplastids

Parasites of the phylum euglenozoa, i.e., the kinetoplastids, are the causative agents of various infections that are classified as neglected tropical diseases. Overall, it is estimated that one billion people in tropical and subtropical countries are affected. Infections with *Leishmania* spp. lead to cutaneous (*Leishmania major, Leishmania tropica*) mucocutaneous (*Leishmania braziliensis*), or visceral leishmaniasis (*Leishmania donovani, Leishmania infantum*), which is spread by sandflies. About 250,000 new cases are registered per year in 87 countries (WHO, 2017a). For treatment, sodium stibogluconate, amphotericin B, miltefosine, paromomycin, and pentamidine are used; yet, the therapy needs major improvement as it is characterized by high levels of toxicity for the patient. Further, resistance against the drugs, in particular to the pentavalent antimonial stibogluconate, strongly limits their usability (Loiseau and Bories, 2006; Ponte-Sucre et al., 2017).

Parasites of a related kinetoplastid species, *Trypanosoma*, cause life-threatening infections, i.e., human African trypanosomiasis or sleeping sickness (*Trypanosoma brucei*) and Chagas disease *(Trypanosoma cruzi*). Human African trypanosomiasis is spread by the tsetse fly in tropical Africa. Approximately 3,000 cases were reported in 2016 (WHO, 2016). In the first hemolytic stage, *T. brucei* replicates extracellularly in the host blood causing fever and joint pain among other symptoms. In the second, severe neurological stage of the disease, the parasite reaches the central nervous system. Patients suffer from disruption of the sleep-wake cycle and irreparable neurological damage. Parasites in the peripheral blood stream can be attacked by the drugs suramin and pentamidine. For the central nervous system form, only the mercurial melarsoprol and eflornithine are available (Brun et al., 2010). As in the case of leishmaniasis, modern, i.e., less toxic and more effective drugs are needed. *T. cruzi*-derived Chagas disease is prevalent in Latin-America claiming 14.000 deaths per year. An estimated 6 million people are infected by *T. cruzi* spread by the bug *Triatoma infestans* (also kissing bug or winchuka). As a treatment, the chemical radical-producing drug benznidazole and nifurtimox are available. With only two compounds at hand, limited success rates and severe side-effects, new drugs are required against this parasite (Castro et al., 2006).

### Amoebae

The free-living amoebozoan parasite *Entamoeba histolytica* causes amoebiasis. With an estimated death toll of 40,000–100,000 per year, it ranges second behind *Plasmodium* infections (Stanley, 2003). The disease is most prevalent in but not restricted to the tropics when sanitation is poor. Although the majority of infections progress asymptomatically, a life-threatening amoebic colitis can manifest. *E. histolytica* forms hardy, infectious cysts that are ingested by the host via contaminated food or water. After reaching the colon, the cysts transform into trophozoites that are capable of invading the intestinal mucosa. When breaching the mucosa, trophozoites can disseminate, among others, to the liver and the central nervous system causing serious complications, i.e., amoebic abscesses (Shirley and Moonah, 2016). First line treatment is a combination of the antibiotics metronidazole and paromomycin. Alternative, second line treatments for metronidazole are other nitroimidazoles, e.g., tinidazole or ornidazole, and the broad-spectrum anti-parasitic nitazoxanide. Paromomycin can be substituted by diiodohydroxyquinoline and diloxanide, both drugs act by a so-far unresolved mechanism and may not be effective against all strains (McAuley and Juranek, 1992).

The severity of infections by protozoan parasites, the limited arsenal of drugs, often with hardly tolerable side effects, and the increasing resistance problem call for novel approaches. The scope of this review is, thus, to discuss the potential of channel and transport proteins as novel targets for anti-parasite chemotherapy in terms of druggability, selectivity, and proneness to resistance. There is considerably more data available from the malaria research field compared to the more neglected parasite-caused diseases. A major criterion for inclusion of a channel or transporter into this review was existing proof of principle involving first small-molecule inhibitors that exhibit anti-parasitic potency.

## Targeting transport processes of parasites at different levels

**Figure 1:**
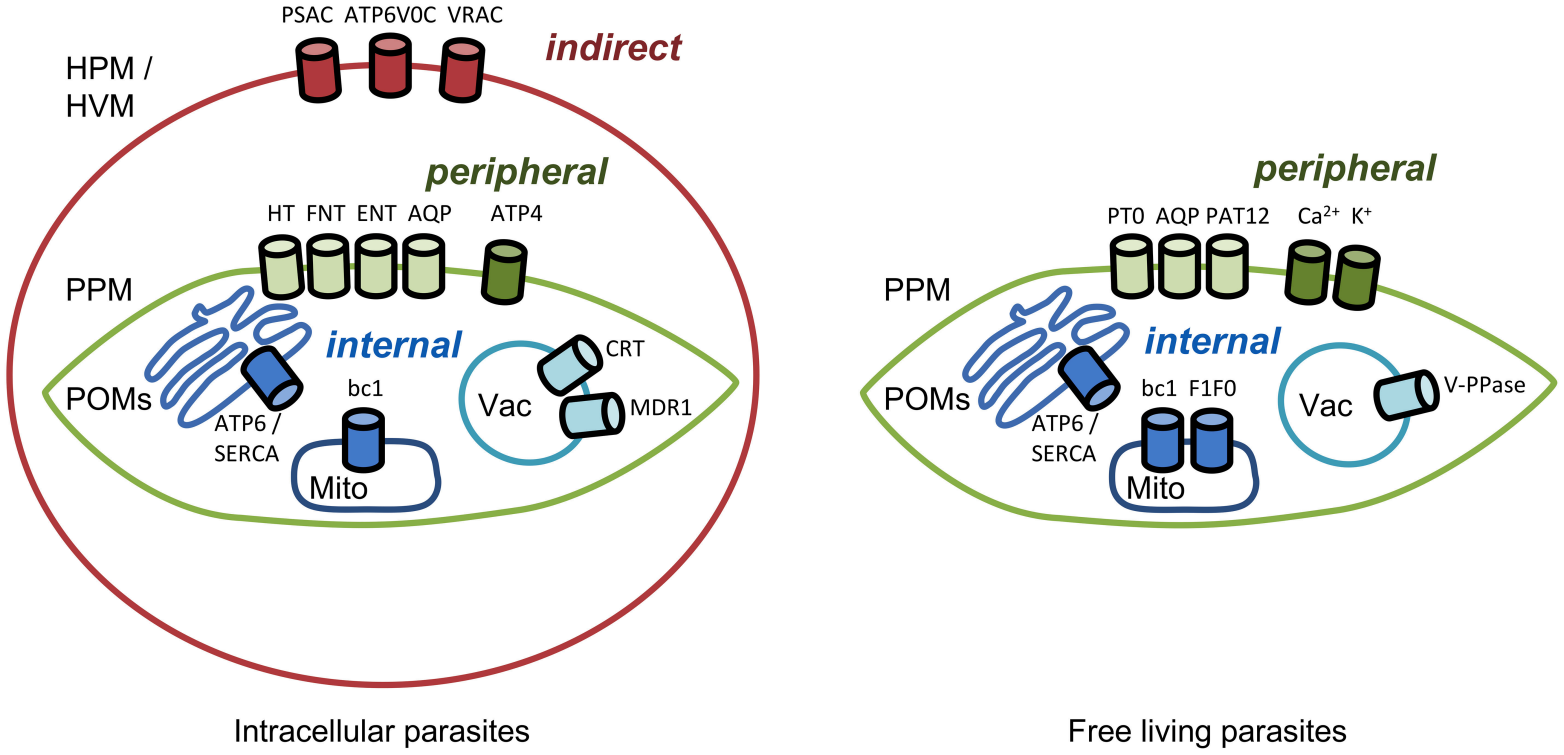
Channels and transporters of parasites as targets for *indirect, peripheral*, and *internal* therapeutic attacks. HPM, host plasma membrane; HVM, host vacuolar membrane; PPM, parasite plasma membrane; POMs, parasite organelle membranes; Mito, mitochondrion; Vac, vacuole. Abbreviations of the channel and transporter proteins are explained in the text.

Transmembrane transporters and channels are usually classified based on their biophysical and biochemical properties, such as mechanism of transport and substrate selectivity. We decided to provide a pharmacological and pharmaceutical view on the topic and structured this manuscript based on the location of the transporter of interest and, accordingly, the site of action of a respective drug (Figure 1). We will approach the parasite from the outside, first hitting the host cell in the case of intracellular parasites. i. *Indirect targeting*. If it is possible to address infected host cells selectively by targeting transport proteins of the host cell plasma membrane this would leave the parasite little options for defending itself against the attack. Malaria parasites for instance are known to modify the functionality of red blood cell proteins and to integrate plasmodial membrane proteins into the erythrocyte membrane. ii. *Peripheral targeting*. Target proteins residing in a parasite's plasma membrane possibly can be inhibited from the outside. In this case and in *indirect* targeting, resistance mechanisms would be limited to changing the drug binding site of the target protein. iii. *Internal targeting*. For targeting transporters within a parasite, i.e., at organelles such as the digestive vacuole or mitochondria, respective drugs would need to enter the parasite's cytosol. In this case, the parasite has additional means to generate resistance, either by preventing uptake of the compound, by altering it chemically, or by pumping it out via efflux transporters. Hence, we will also address iv. *Targeting drug efflux transporters*.

### Indirect targeting—channels and transporters of the infected host cell membrane

It was recognized early on in malaria research that the transport of ions, amino acids, and other nutrients across the plasma membrane of infected red blood cells increases compared to uninfected cells. This way, the parasite actively adapts the ionic environment inside the erythrocyte to its needs and ensures access to nutrients from the host blood. Over the years, it has become evident that such new permeability pathways (NPP) are not only due to infection-dependent alteration of the host membrane proteins but also to export of *Plasmodium*-derived proteins and integration into the host cell membrane (Overman, 1947; Ginsburg et al., 1985; Desai et al., 2000; Huber et al., 2002). Proteins at the erythrocyte plasma membrane pose attractive targeting sites as the respective inhibitor compounds would not be in direct contact with the parasite. Interference by the parasite with drug action would be restricted to alteration of the protein resulting from gene mutations, whereas other means, such as expedited drug export or metabolism would not be applicable. It remains to be shown, however, whether this *indirect* approach can reliably kill parasites (Cohn et al., 2003). The efficiency of compounds that indirectly target parasites is summarized in Table 1.

**Table 1:** Efficiency of compounds for *indirect* targeting.

**Target**	**Parasite species**	**Compound name**	**Effect on protein**	**Effect on parasite**	**Cell stage *in vitro***	**Effect *in vivo***	**Host species**	**Reference**
PSAC	*P. falciparum*	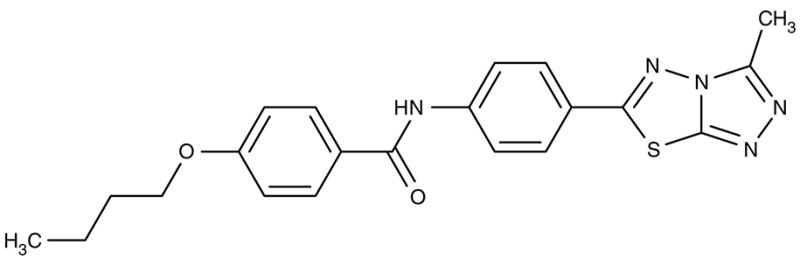 PRT1-20	–	IC_50_ 5 μM	Trophozoites	–	Human	Pain et al., 2016
		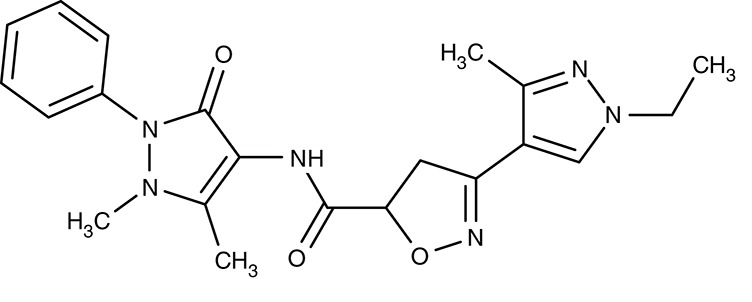 ISPA-28	–	IC_50_ 0.06 μM	Trophozoites	–	Human	Nguitragool et al., 2011
VRAC (host)	*P. berghei*	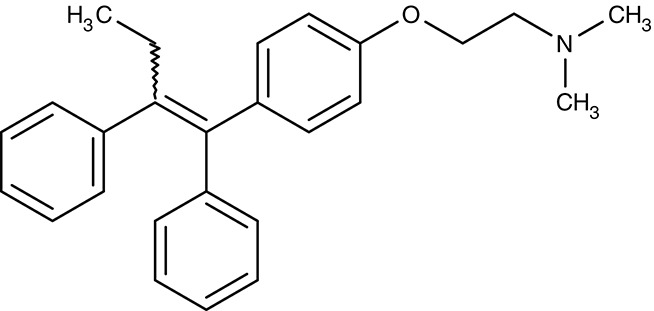 Tamoxifen	IC_50_ 4 μM	–	Liver-stage	–	Human	Prudêncio et al., 2009
		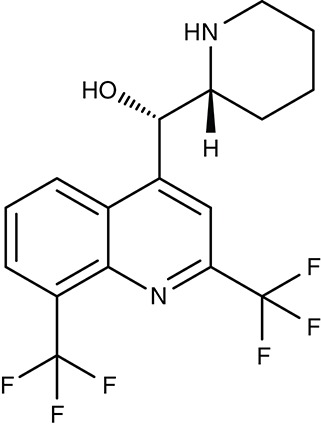 Mefloquine	IC_50_ 2 μM	–	Liver-stage	–	Human	Prudêncio et al., 2009
ATP6V0C (host)	*L. donovani*	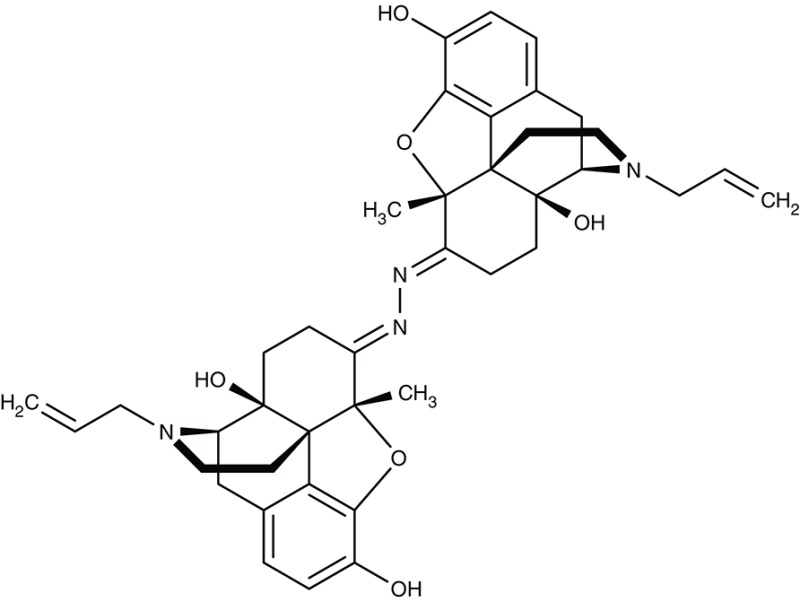 Naloxonazine	–	IC_50_ 3.5 μM	Amastigotes (Intracellular)	–	Human	de Muylder et al., 2011

#### Plasmodial surface anion channel

One extensively studied type of conductivity of the red blood cell membrane that is brought about upon infection is derived from the plasmodial surface anion channel (PSAC; Figure 1). Despite a still elusive protein identity, permeability for various substrates, such as sugars, amino acids, nucleosides, and inorganic anions and cations, has been attributed to this voltage-dependent channel (Ginsburg et al., 1985; Kirk and Horner, 1995; Upston and Gero, 1995; Saliba et al., 1998; Hill et al., 2007). PSAC seems to transport the mentioned substrates via two different routes within the protein or protein complex. One path is said to be used primarily for alanine and sorbitol uptake and can be blocked by furosemide, whereas the other one conducts mainly proline and the unnatural substrate phenyltrimethylammonium (PhTMA). The latter path is sensitive to so-called PSAC residual transport inhibitors, abbreviated as PRT (Alkhalil et al., 2004; Pain et al., 2016). Based on this finding, full blockade of PSAC may require a combination of a PRT inhibitor plus a furosemide derivative. For example, PRT1-20 alone (Table 1) yielded an IC_50_ on *P. falciparum* growth of 5 μM; in combination with furosemide it was 10 times lower (Pain et al., 2016). Toward identification of the PSAC protein or regulators thereof, the finding could become helpful that certain parasite strains are susceptible to one particular group of PSAC inhibitors. This pointed to two genes, CLAG3.1 and CLAG3.2 (cytoadherence linked asexual protein) of which only one appears to be active at a time. Mutations in the genes led to modified PSAC activity. Further, epigenetic regulation of the CLAG3 genes was suggested to modulate PSAC (Sharma et al., 2013; Nguitragool et al., 2014). To illustrate this, ISPA-28 (Table 1), an isolate-specific PSAC antagonist, exhibited an IC_50_ of 56 nM against the *P. falciparum* Dd2 strain and 43 μM against the HB3 strain, i.e., a value higher by three orders of magnitude. When the CLAG3 gene of the Dd2 strain was transferred to the HB3 strain, it showed the same nanomolar susceptibility (Nguitragool et al., 2011, 2014). Clearly, identification of the true nature of the PSAC protein and/or components as well as expression or reconstitution in a heterologous or artificial system would be highly appreciated for in-depth structure-function analyses, inhibitor screening and development.

#### Host ion channels

Besides the parasite-derived PSAC, host encoded transport proteins of the erythrocyte may be exploited as drug targets if their functionality changes with infection. Oxidative stress was shown to alter potassium and chloride conductance of infected red blood cells (Staines et al., 2001; Huber et al., 2004). However, specific inhibitors are yet to be found to determine their potential as anti-parasite drugs.

There is evidence that in the liver-stage, *Plasmodium berghei* infection leads to a sevenfold increase in chloride conductance of the host's volume-regulated anion channel, VRAC, as found using a human hepatoma cell line (Figure 1; Prudêncio et al., 2009). Conductivity was inhibited by tamoxifen, and mefloquine at single-digit micromolar concentrations (Table 1). The underlying mechanism of channel activation by the parasite, the effect of estrogen receptor modulators on VRAC conductivity, and, ultimately, whether indirect targeting of VRAC would be suitable for malarial therapy of the liver stage is not clear at this time. With the system and compounds at hand further investigations will be possible that may shed some light on the phenomenon.

#### Host V-type proton ATPase

In the search of new drug targets against *L. donovani*, Muylder et al. chose a strategy of host-directed therapy. The amastigote form of the parasite develops primarily inside phagocytic cells and is inert to digesting enzymes. A screening assay using a human macrophage cell line infected with *L. donovani* yielded one hit compound, a μ-opioid receptor antagonist naloxonazine (de Muylder et al., 2011; Table 1). It turned out that naloxonazine upregulates expression of the V-type proton ATPase subunit C, ATP6V0C. This upregulation was linked to an increase in the volume of intracellular acidic vacuoles suggesting an *indirect* effect on *Leishmania* amastigotes through host cell vacuolar remodeling (de Muylder et al., 2016). How such a therapy would be tolerated by the host and whether the parasites will find ways to adapt to the remodeled vacuoles is not known.

#### Host nutrient channels and transporters

We describe two examples illustrating that nutrient transport of the host cell affects growth of *P. berghei* parasites, i.e., in the blood-stage depending on the glycerol permeability of an aquaporin (Liu et al., 2007), and in the liver-stage via arginine transport (Meireles et al., 2017). Aquaporin-9 knockout mice lack a functional glycerol channel in their erythrocytes. In this environment, *P. berghei*, grew considerably slower compared to wildtype erythrocytes, and infected AQP9-null mice survived longer. The authors attribute the effect to reduced glycerol levels in the parasite impeding glyceroplipid biosynthesis for the build-up of membranes during growth. Similarly, knockdown of the arginine-transporting SLC7A2 of the solute carrier family decreased intra-hepatic growth and multiplication of *P. berghei* parasites *in vivo* and *ex vivo* (Meireles et al., 2017). A sufficient supply of arginine is required for the vital polyamine synthesis of the parasites. Today, glycerol or arginine transport-modulating small molecules have not been found and/or tested.

Together, although an *indirect* approach holds strong potential against parasite infections, several gaps in basic knowledge need to be filled with regard to the identity of the involved transport proteins, selectivity of inhibitors, and susceptibility/adaptability of the parasites.

### Peripheral targeting—channels and transporters of the parasite plasma membrane

The substrate spectrum of the parasite-induced new permeation pathways at the host cell membrane is broad. Transport proteins at the parasite's plasma membrane (Figure 1), in turn, appear much more specific. These membrane proteins facilitate the uptake of the main energy source, glucose, and precursors for biosynthesis, such as nucleosides for DNA/RNA, or glycerol for glycerolipids. Equally vital is the efficient release of waste molecules derived from energy metabolism, e.g., lactic acid, or from protein degradation, i.e., ammonia and urea. Nutrient and metabolite transport often depends on transmembrane ion gradients, e.g., of protons or sodium, generated by ATPases and is further modulated by ion channels. Efficiency data on compounds for peripheral parastite targeting are displayed in Table 2.

**Table 2:** Efficiency of compounds for *peripheral* targeting.

**Target**	**Parasite species**	**Compound name**	**Effect on protein**	**Effect on parasite**	**Cell stage *in vitro***	**Effect *in vivo***	**Host species**	**Reference**
HT	*Plasmodium* spp.	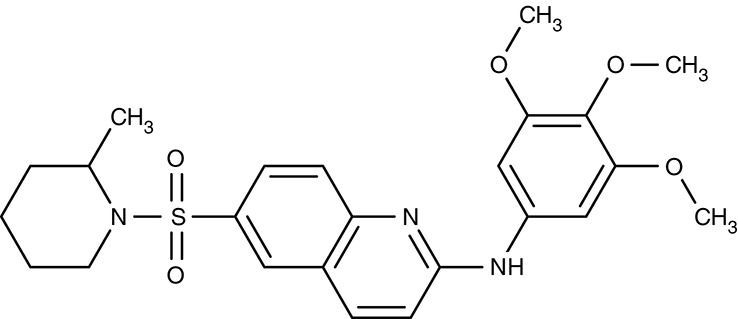 TCMDC-125163	IC_50_ 39 nM	IC_50_ 1.24 μM	Trophozoites	–	–	Ortiz et al., 2015
		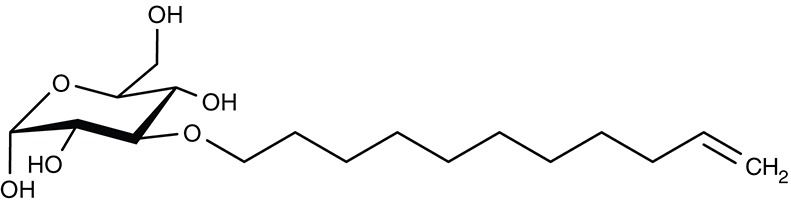 C3361	K_i_ 8.6–53 μM	IC_50_ 15–16 μM	Trophozoites	–	–	Joet et al., 2003; Blume et al., 2011
			–	IC_50_ 11 μM	Liver-stage	–	Human	Slavic et al., 2011
HT1	*B. bovis*		K_i_ 4.1 μM	No inhibition at 100 μM	Trophozoites	–	–	de Muylder et al., 2011
GT1	*T. gondii*		K_i_ 82 μM	No inhibition at 200 μM	Tachyzoites	–	–	Blume et al., 2011
FNT	*P. falciparum*	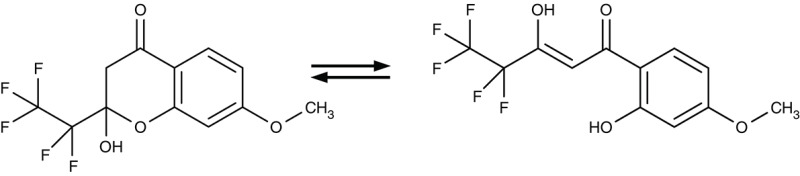 MMV007839	IC_50_ 0.02–0.17 μM	IC_50_ 0.14 μM	Trophozoites	–	–	Golldack et al., 2017; Hapuarachchi et al., 2017
		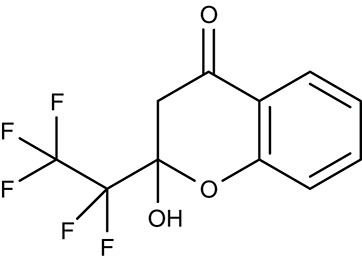 MMV000972	IC_50_ 0.05–0.17 μM	IC_50_ 1.70 μM	Trophozoites	–	–	
PT0	*T. brucei*	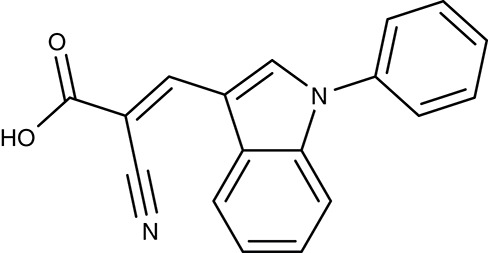 UK5099	Inhibition at 250 μM	–	–	–	–	Sanchez, 2013
PAT12	*T.cruzi*	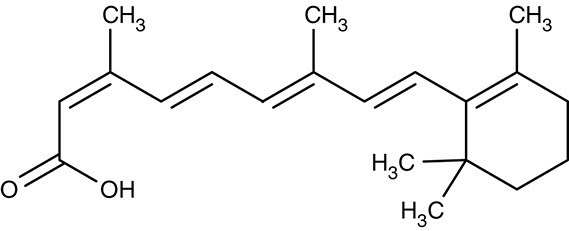 Isotretinoin	–	IC_50_ 0.13 μM	Epimastigotes	–	–	Reigada et al., 2017
				IC_50_ 30.6 μM	Trypomastigotes	–	–	
ENT1	*Plasmodium* spp.	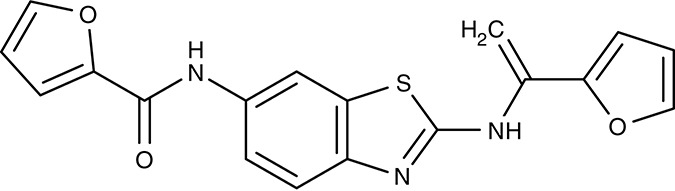 ChemBridge 9001893 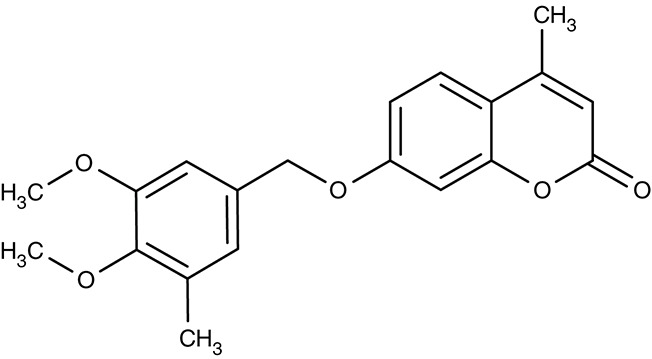 ChemBridge 6946484	IC_50_ 2.5–30 nM	IC_50_ 3–55 μM	Trophozoites	–	–	Frame et al., 2015b
ATP4	*P. falciparum*	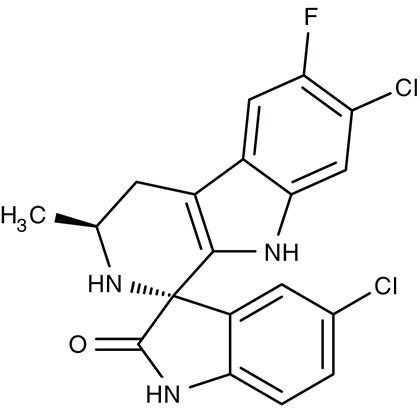 Cipargamin	–	IC_50_ 0.5–1.4 nM	Trophozoites	Clearance with 3-day dosing, 30 mg per day	Human	Rottmann et al., 2010; Spillman and Kirk, 2015
		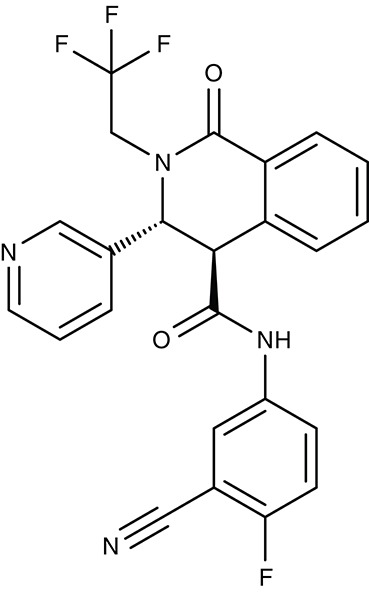 SJ733	–	IC_50_ 30 μM	Trophozoites	–	–	Spillman and Kirk, 2015
Calcium channels	*Leishmania* spp. *Trypanosoma* spp.	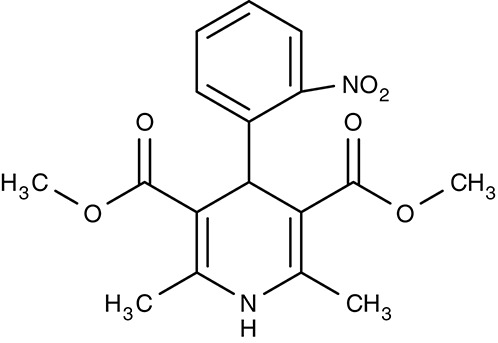 1,4-dihydropyridines (e.g., Nifedipine)	−−	IC_50_ 2.6–181 μM	Promastigotes/Amastigotes Trypomastigotes	−−	−−	Tempone et al., 2009 Reimão et al., 2010, 2011
	*A. castellanii*	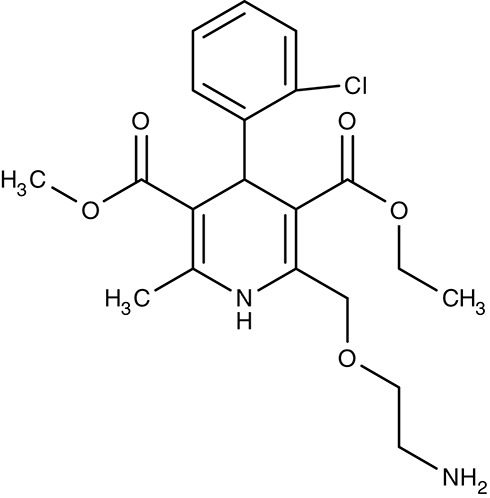 Amlodipine	–	Large inhibition at 1.2 μM	Trophozoites	–	–	Baig et al., 2013
	*L. infantum*	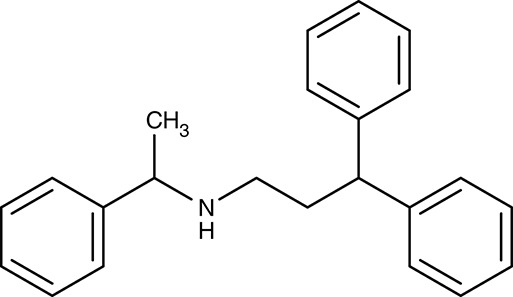 Non-dihydropyridines (e.g., fendiline)	–	IC_50_ 2–16 μM	Promastigotes	–	–	Reimão et al., 2016
	*T. cruzi*		–		Epimastigotes	–	–	Reimão et al., 2016
K1/K2	*T. brucei*	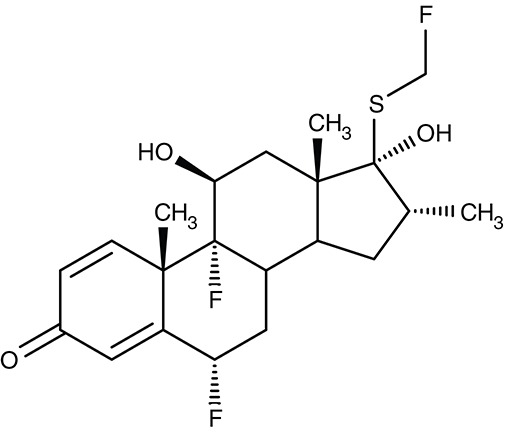 Fluticasone	IC_50_ 0.7 μM	–	–	–	–	Schmidt et al., 2017

#### Targeting peripheral nutrient and metabolite transporters

Considering their significance for survival, it seems quite surprising that plasmodia rely on a single hexose transporter, HT, and a single lactic acid transporter, the latter being a member of the microbial formate-nitrite-transporter family, FNT. Both transporters are present at the plasma membrane and both have been validated as novel antimalarial drug targets using cultured parasites.

##### Glucose transporters

Soon after the identification of HT, first weak glucose-analog inhibitors were described (Krishna and Woodrow, 1999; Woodrow et al., 1999; Joet et al., 2003). One of these compounds, C3361 (Table 2), yielded K_i_-values in the μM range on glucose transport of *Plasmodium berghei, Plasmodium falciparum, Plasmodium yoelii, Plasmodium vivax, Plasmodium knowlesi, Babesia bovis*, and *T. gondii* (Joet et al., 2003; Blume et al., 2011). C3361 was not only active in the blood-stage but also inhibited parasite development in the liver-stage of *P. berghei* with an IC_50_ of 11 μM (Slavic et al., 2011). The vector stages, however, were much less susceptible, and a transmission block required 1 mM. Interestingly, C3361 failed to inhibit growth of the related apicomplexan *Babesia* parasites suggesting an alternative glucose transport pathway. In fact, the *B. bovis* genome contains two putative hexose transporter genes, of which only one has been characterized so-far (Derbyshire et al., 2008). Knockout of the homologous *Toxoplasma* glucose transporter, GT, led to moderate growth inhibition. Apparently, it is dispensable for the survival of the parasite. A search for alternative transporters in *Toxoplasma* produced three more putative sugar transporters of which one was found to be located at the plasma membrane. Yet, a knockout failed to effect parasite growth. Contrary to plasmodia, *Toxoplasma* seems not to rely exclusively on glucose as an energy source. It is discussed that glutamine can be used as a potent alternative energy source (Blume et al., 2009).

More recent screenings for inhibitors of the plasmodial HT using the Tres Cantos antimalarial compound set (TCAMS) and the malaria box led to the discovery of nanomolar inhibitors, e.g., TCMDC-125163 (Table 2) has an IC_50_ of 39 nM for heterologously expressed HT, 3.2 μM for the human red blood cell glucose transporter GLUT1, and an EC_50_ of 1.24 μM for growth of cultured *P. falciparum* parasites. Binding of the identified compounds occurred mostly non-competitive with glucose and, hence, likely to a site different from the glucose binding pocket (Ortiz et al., 2015).

##### Lactate and pyruvate transporters

The end products of glucose-based energy metabolism are lactic acid in plasmodia, and pyruvic acid in trypanosomes. In order to prevent detrimental acidification of the cytosol and inhibition of the metabolic pathway by accumulating product, such molecules need to be swiftly released from the cells. Lactate transport in living *P. falciparum* parasites was experimentally shown in the early 1990s (Kanaani and Ginsburg, 1991). It took until 2015 that the responsible transporter was identified by our and Kiaran Kirk's group (Marchetti et al., 2015; Wu et al., 2015). The protein is structurally and in terms of transport mechanism unrelated to human lactate transporters from the monocarboxylate transporter family (MCT). Instead, the plasmodial lactic acid transporter is a member of the microbial formate-nitrite transporter family, FNT. Besides l-lactate, it transports d-lactate, as well as formate, acetate and pyruvate by a proton cotransport mechanism (Wiechert and Beitz, 2017; Wiechert et al., 2017).

Screening of the malaria box yielded two compounds, MMV007839 and MMV000972 (Table 2), that efficiently block PfFNT at nanomolar concentrations (Spangenberg et al., 2013; Golldack et al., 2017). *In vitro* selection of a resistant *P. falciparum* strain helped to locate the binding site at the intracellular face of the transporter. The compounds, thus, need to enter the parasite where they assume a lactate substrate-like form carrying a negative charge for efficient binding. Transport across consecutive membranes that shield the parasite is achieved by a cyclic hemiketal form that is neutral and lipophilic, see Table 2 (Golldack et al., 2017). FNTs are absent in humans, however, some other protozoan parasites carry single or multiple copies of FNT genes, e.g., *Babesia* spp., *T. gondii*, and *E. histolytica*, representing putative targets. Kinetoplastids, in turn, do not encode FNTs in their genomes, raising the question of how monocarboxylate transport is achieved in these organisms. In *T. brucei*, a high-affinity pyruvate transporter, TbPT0, was recently discovered that is more related to polytopic proteins from plants than to mammalian MCTs (Sanchez, 2013). This transporter at the plasma membrane plus two additional mitochondrial pyruvate transporters of *T. brucei* were found to be inhibitable by the pyruvate-reminiscent compound UK5099 (Štáfková et al., 2016; Table 2).

##### Nucleobase and nucleoside transporters

Apart from nutrients and metabolites of energy metabolism, precursors, and components of biosynthetic pathways are typical substrates of parasite transporters. In this sense, a group of transporters found at the plasma membrane of plasmodia imports nucleobases and nucleosides. Four *P. falciparum* genes encode equilibrative nucleoside transporters, ENT1–4 of which ENT1 seems to provide the major uptake route (Downie et al., 2006, 2008, 2010; Frame et al., 2012, 2015a). Small molecule inhibitors were found by high throughput screening, e.g., ChemBridge no. 9001893 and 8946464 (Table 2), that inhibited the *P. falciparum* PfENT1 with IC_50_ values in the low nanomolar range. Efficiency was similar with the *P. vivax* and *P. berghei* ENT1 proteins (Arora et al., 2016; Deniskin et al., 2016). The compounds were less potent, however, in parasite cultures with EC_50_-values from 0.8 to 6.5 μM (Frame et al., 2015b).

##### Aquaporin solute channels

*Plasmodium* and *Toxoplasma* parasites express a single aquaglyceroporin channel, AQP, at the plasma membrane (Hansen et al., 2002; Pavlovic-Djuranovic et al., 2006). These AQPs conduct water and small, uncharged solutes that are relevant in glycerolipid biosynthesis (glycerol), protein degradation (urea, ammonia), and oxidative stress (hydrogen peroxide) (Hansen et al., 2002; Beitz et al., 2004; Zeuthen et al., 2006; Wu et al., 2010; Wree et al., 2011; Almasalmeh et al., 2014). Small, drug-like inhibitors for apicomplexan AQPs are missing (Song et al., 2012), but their potential as drug targets is underscored by a *P. berghei* PbAQP knockout strain that exhibited strongly reduced growth, virulence, and progression through the liver stage (Promeneur et al., 2007, 2018). *T. brucei* expresses three AQPs of which TbAQP2 is a key factor for the uptake of the anti-trypanosomal drug pentamidine (Uzcategui et al., 2004; Song et al., 2016). The *L. major* AQP facilitates uptake of antimonite into the parasite released from the anti-leishmanial drug stibogluconate (Mukhopadhyay and Beitz, 2010; Mukhopadhyay et al., 2011).

##### Drug repurposing/polyamine transporters

An attempt to repurpose already used drugs revealed that retinoids, an established class for the pharmacotherapy of severe acne, target parasitic nutrient transporters. Initially, retinoic acid and retinol acetate were shown to inhibit the growth of *L. donovani* (Mukhopadhyay and Madhubala, 1994). More specifically, isotretinoin (Table 2) was found to block a polyamine transporter, PAT12, when adding to cultures of *T. cruzi* epimastigotes. *In vitro* growth of emerging trypomastigotes and epimastigotes was inhibited with IC_50_ values of 0.13 and 30.6 μM, respectively. PAT12 is a member of the polyamine and amino acid transporter family, AAAP, for which isotretinoin displayed activity as a multi-target inhibitor (Reigada et al., 2017). This shows that repurposing is a valid tool and can promote research in the field of neglected diseases.

#### Targeting peripheral ion transporters and channels

The establishment and maintenance of ion gradients across the parasite plasma membrane is vital for the membrane potential, osmotic balance, as well as for driving transport processes. P-type ATPases are single protein units that convert energy from ATP hydrolysis into cation transport (Weiner and Kooij, 2016). ATP4 of *P. falciparum* was recently shown to act as a sodium pump at the plasma membrane (Dyer et al., 1996; Spillman et al., 2013). There is evidence that sodium export by ATP4 is coupled to proton import. Whether cell death upon blockade of ATP4 occurs due to cytosol acidification, osmotic swelling, collapse of the electrochemical potential, or a combination thereof is unknown (Spillman et al., 2013; Spillman and Kirk, 2015). For whatever reason, blocking of ATP4 is lethal for malaria parasites rendering ATP4 a most attractive novel drug target. Analysis of the 400 malaria box compounds yielded the surprisingly high number of 28 hits, which further underscores the central role of ATP4 for parasite viability.

##### ATP4/P-type sodium ATPase

Two previously identified ATP4 inhibitors already entered the clinical trial stage. The clinical candidate cipargamin with a spiroindolone scaffold (Table 2) is thought to bind to the transport path of ATP4 from the intracellular entry site as deduced from *in vitro* selection of resistance mutations (Spillman and Kirk, 2015). Growth of sensitive *P. falciparum* strains was inhibited with IC_50_-values in the range of 0.5–1.4 nM. Application of a single 100 mg kg^−1^ dose in an *in vivo* mouse model study killed all *P. berghei* parasites. In human trials, a 3-day dosing regime with 30 mg per day led to parasite clearance (Rottmann et al., 2010; White et al., 2014). Cipargamin exhibited low toxicity in humans, high oral bioavailability and suitable half-life. Other related ATP4-inhibiting spiroindolones have been found to be similarly potent (Spillman et al., 2013). The second promising candidate undergoing a clinical trial is the dihydroisoquinolone SJ733 (Jiménez-Díaz et al., 2014; Table 2). It shows more distant structure similarities to cipargamin with the 5-membered heterocycle of the indolone moiety replaced by a 6-membered ring (Jiménez-Díaz et al., 2014; Spillman and Kirk, 2015; Crawford et al., 2017). Although resistance mutations were selectable *in vitro* by sub-lethal concentrations, ATP4 inhibitors, when dosed properly, might prove advantageous against the rise of resistant strains in the clinic due to their fast acting property.

##### Drug repurposing/calcium channels

Drug repurposing approaches aim at ion channels at the plasma membrane of kinetoplastids. Several established 1,4-dihydropyridine calcium channel blockers used for the treatment of hypertension in humans were tested on various *Leishmania* and *Trypanosoma* species. Nifedipine, amlodipine, bepridil, nimodipine, and others showed weak effects *in vitro* with IC_50_-values in the micromolar range (Maya et al., 2000; Tempone et al., 2009; Reimão et al., 2010, 2011). Amlodipine and lacidipine administered in four weekly single doses of 10 mg kg^−1^ reduced the parasite burden of *L. donovani*-infected BALB/c mice by 75–85% (Palit and Ali, 2008). Amlodipine was also tested for activity against cultured amoebae of *Acanthamoeba castellanii* and largely inhibited growth at 1.2 μM (Baig et al., 2013). The non-dihydropyridine calcium channel blockers fendiline, mibrefadil, and lidoflazine inhibited *in vitro* growth of *L. infantum* promastigotes and *T. cruzi* epimastigotes with IC_50_-values from 2–16 μM (Reimão et al., 2016). Verapamil, however, failed to inhibit growth of *L. donovani* promastigotes, but seemed to reverse the resistance against stibogluconate by an unknown mechanism (Neal et al., 1989; Valiathan et al., 2006). Although being calcium channel blockers in humans, the target in the tested parasites remains to be established.

##### Drug repurposing/potassium channels

Recently, a screening by the National Center for Advancing Translational Sciences Small Molecule Resource identified fluticasone, an established corticosteroid for the treatment of asthma, to inhibit the *T. brucei* potassium channels TbK1 and TbK2. The proteins were localized to the parasite's plasma membrane and electrophysiologically characterized in *Xenopus* oocytes (Steinmann et al., 2015). Fluticasone was found to inhibit the TbK1/TbK2-mediated currents at an IC_50_ of 0.7 μM (Schmidt et al., 2017).

### Internal targeting—channels and transporters of parasite organelle membranes

Drugs that need to enter the parasite's cytosol encounter several more challenges than compounds acting from the outside. Diffusional uptake across the plasma membrane requires high lipophilicity, small molecule size, and absence of charged moieties. Alternatively, compounds can be shuttled into the cell by a more active type of transport via endogenous channels and transporters. This route is taken for instance by antimonite released from stibogluconate in *Leishmania* therapy or by pentamidine against trypanosomes. In both cases, resistance mutations of a transporting aquaglyceroporin efficiently prevent the drugs from entering the parasites. Another factor is the metabolic stability of the drug within the parasite cell. This area is not well studied, yet it is conceivable that inactivation by chemical modification may well occur in a similar fashion as in the host cells, which detoxify xenobiotics e.g., by oxidation and conjugation reactions. Finally, drugs that actually made it to the site of action in a functional form may be pumped out of the parasite cell by drug resistance transporters. The prominent example is the chloroquine resistance transporter, CRT. Table 3 gives an overview on the new developments of compound targeting channels and transporters of internal organelle membranes.

**Table 3:** Efficiency of compounds for *internal* targeting.

**Target**	**Parasite species**	**Compound name**	**Effect on protein**	**Effect on parasite**	**Cell stage *in vitro***	**Effect *in vivo***	**Host species**	**Reference**
ATP6/SERCA	*P. falciparum*	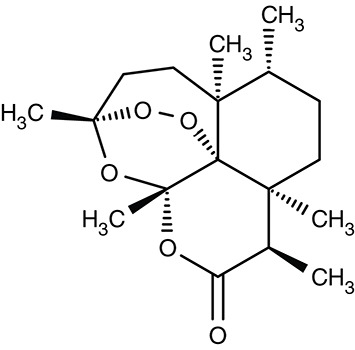 Artemisinin	–	IC_50_ 11 – 13 nM	Trophozoites	–	–	del Pilar Crespo et al., 2008
	*T. gondii*		Inhibition at 10 μM	IC_50_ 0.36–8 μM	Tachyzoites	–	–	Berens et al., 1998; Jones-Brando et al., 2006; Nagamune et al., 2007; Hencken et al., 2010
	*Trypanosoma* spp.		–	IC_50_ 13–20 μM	Trypomastigotes/Epimastigotes	–	–	Yang and Liew, 1993; Mishina et al., 2007; Sen et al., 2010
	*Leishmania* spp.		–	IC_50_ 0.75–120 μM	Promastigotes/Amastigotes	Reduction of parasite burden with oral dose of 10 mg kg^−1^	Mouse/Hamster	
	*B. gibsoni*		–	IC_50_ 2.2 μM	Trophozoites	–	–	Iguchi et al., 2015
	*P. falciparum*	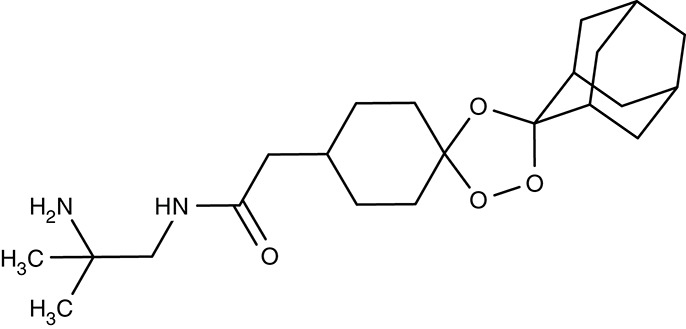 Arterolane	K_i_ 7.7 μM	IC_50_ 1.5 nM	Trophozoites	–	–	Abiodun et al., 2013
	*P. falciparum*	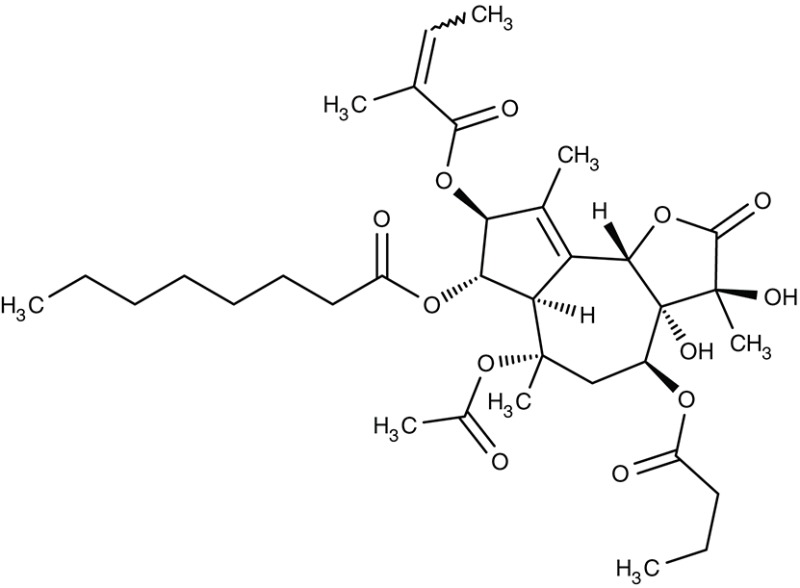 Thapsigargin	–	IC_50_ 0.25– 0.30 μM	Trophozoites	–	–	del Pilar Crespo et al., 2008; Abiodun et al., 2013
	*T. gondii*		Inhibition at 1 μM	–	–	–	–	Nagamune et al., 2007
	*Trypanosoma* spp.		–	IC_50_ 30–34 μM	Trypomastigotes/Epimastigotes	–	–	Mishina et al., 2007
	*L. donovani*		–	28.1 μM	Promastigotes	–	–	Mishina et al., 2007
	*E. invadens*		–	Inhibition of encystation at 0.5 μM	Trophozoites	–	–	Martínez-Higuera et al., 2015
	*N. canium*		–	Growth Inhibition at 0.1 μg ml^−1^	Tachyzoites	–	–	Kim et al., 2002
Cytochrome bc_1_ complex	*P. falciparum*	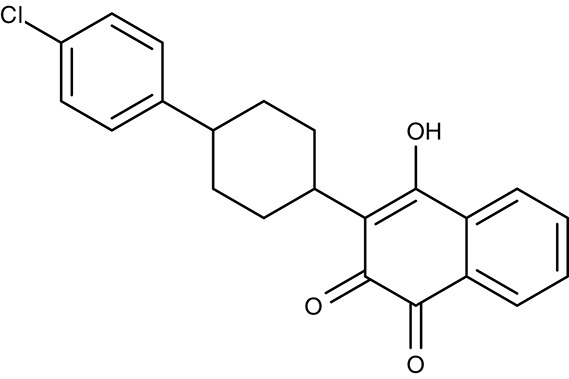 Atovaquone	IC_50_ 0.2 nM	IC_50_ 2 nM	Trophozoites	–	–	da Cruz et al., 2012
	*T. gondii*		–	IC_50_ 0.1–0.5 μM	Tachyzoites	IC_50_ 0.14–0.85 μM	Mouse	Doggett et al., 2012
	*Babesia* spp.		–	IC_50_ 94 nM	Trophozoites	Effective at dose of 100 mg kg^−1^ d^−1^	Hamster	Hughes and Oz, 1995; Wittner et al., 1996; Matsuu et al., 2008
			–	–	–	Effective at dose of 1500 mg d^−1^ plus azithromycin 500 mg on day 1 and 250 mg per day thereafter	Human	Krause et al., 2000
	*L. donovani*		–	–	–	30 % reduction of parasite burden with 100 mg kg^−1^ for 5 days	Mouse	Croft et al., 1992
	*Theileria* spp.	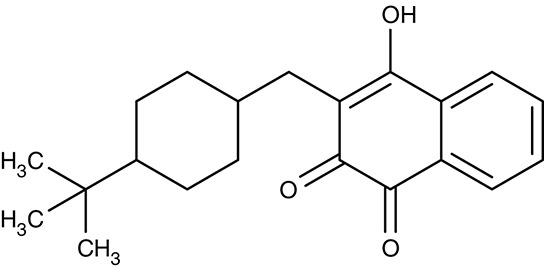 Buparvaquone	–	–	–	Effective at 2.5–6 mg kg^−1^	Cattle, Horse	McHardy et al., 1985; Zaugg and Lane, 1989; Muraguri et al., 1999; Mhadhbi et al., 2010
	*Leishmania* spp.		–	IC_50_ 0.001–5.495 μM	Promastigotes/Amastigotes	60% reduction of parasite burden with 100 mg kg^−1^ for 5 days	Mouse	Croft et al., 1992; Mäntylä et al., 2004a; Reimão et al., 2012; Jamal et al., 2015
	*P. falciparum*	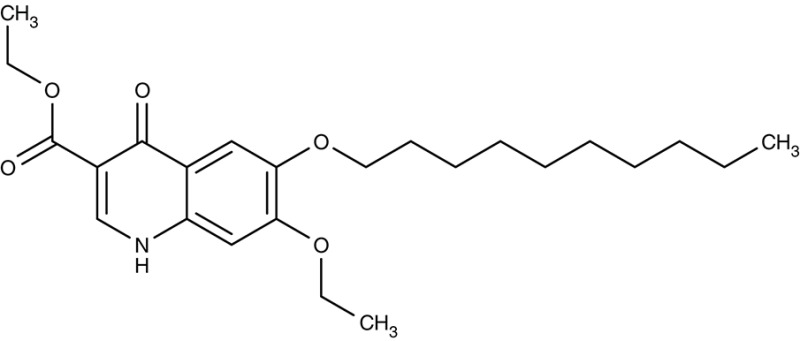 Decoquinate	IC_50_ 2 nM	IC_50_ 2.6–36 nM	Trophozoites	–	–	da Cruz et al., 2012
	*P. falciparum*	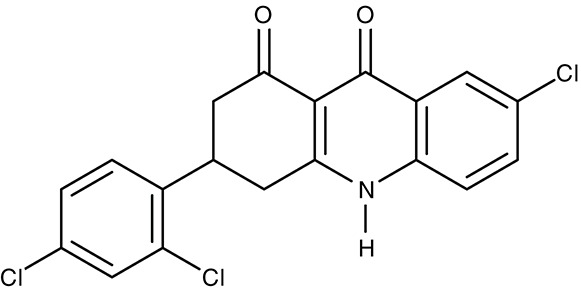 WR249685	–	IC_50_ 3 nM	Trophozoites	–	–	Biagini et al., 2008
	*P. falciparum*	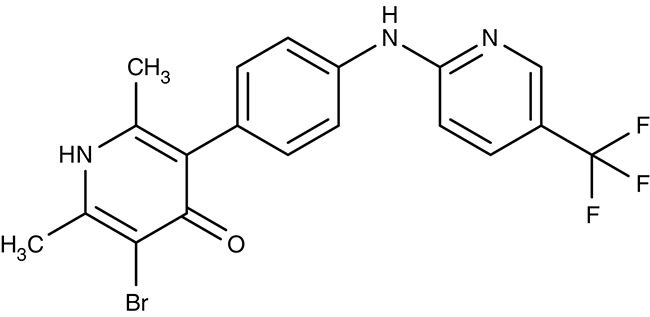 TCMDC-135546	–	IC_50_ 22 nM	Trophozoites	–	–	Raphemot et al., 2015
Mitochondrial F1F0 ATPase	*T. brucei*	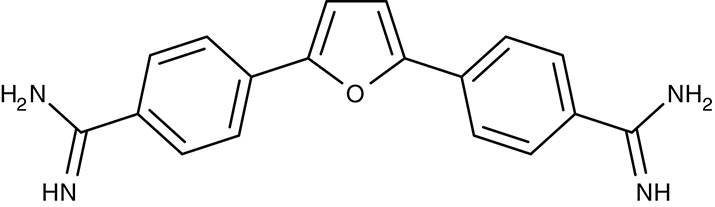 Furamidine (DB75)	Inhibition at 10 μM	IC_50_ 4.5 nM	Trypomastigotes	Effective as pentamidine at 3 mg kg^−1^ d^−1^, for 7 days	Gerbil	Steck et al., 1982; Ismail et al., 2003; Lanteri et al., 2008
		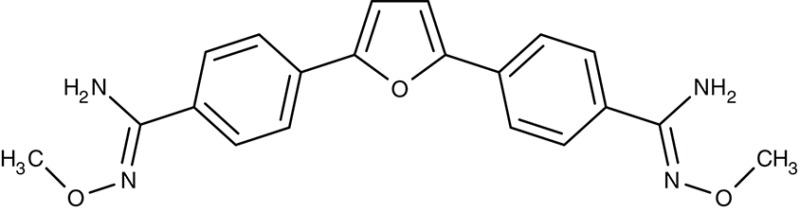 Pafuramidine (DB289)	–	IC_50_ 14.6 μM	Trypomastigotes	Effective at a dose of 400 mg kg^−1^ p.o.	Mouse	Ansede et al., 2004
		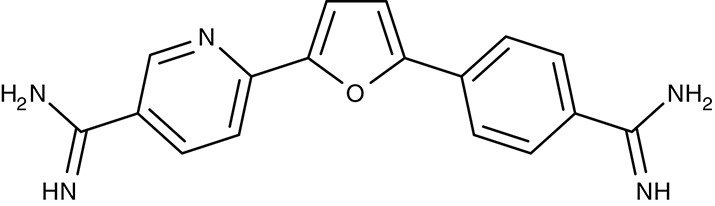 DB820	–	IC_50_ 7.9–141 nM	Trypomastigotes	Effective at 10 mg kg^−1^ i.p.	Mouse	Wenzler et al., 2009
		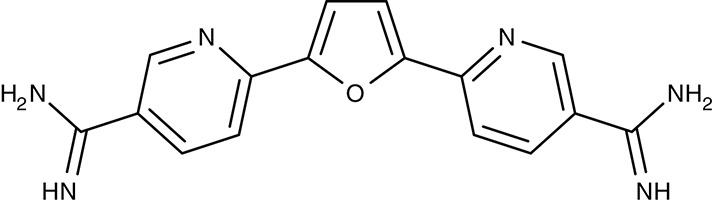 DB829	–	IC_50_ 20–346 nM	Trypomastigotes	Effective at 10 mg kg^−1^ i.p.	Mouse	Wenzler et al., 2009
Mitochondrial choline transporter	*P. falciparum*	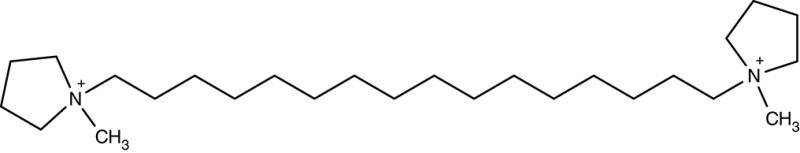 G25	–	–	–	–	–	Wengelnik et al., 2002; Biagini et al., 2004
	*T. brucei*		–	EC_98_ 0.16 μM	Trypomastigotes	–	–	de Macêdo et al., 2015
VPPase VP1	*T. gondii*	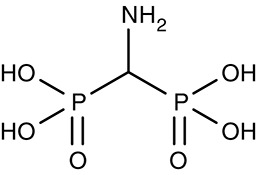 AMDP	IC_50_ 0.9 μM	Inhibition at 5–10 μM	–	–	–	Drozdowicz et al., 2003
CRT	*P. falciparum*	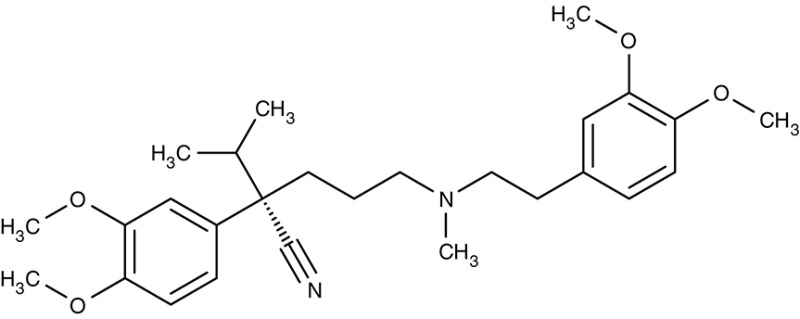 Verapamil	IC_50_ 30 μM	–	–	–	–	Ye and van Dyke, 1994
		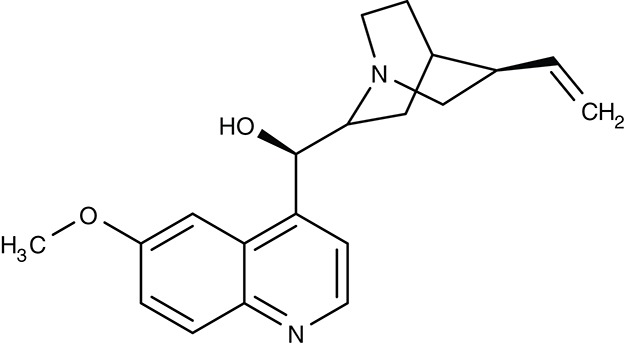 Quinine	IC_50_ 48 μM	–	–	–	–	Martin et al., 2009
		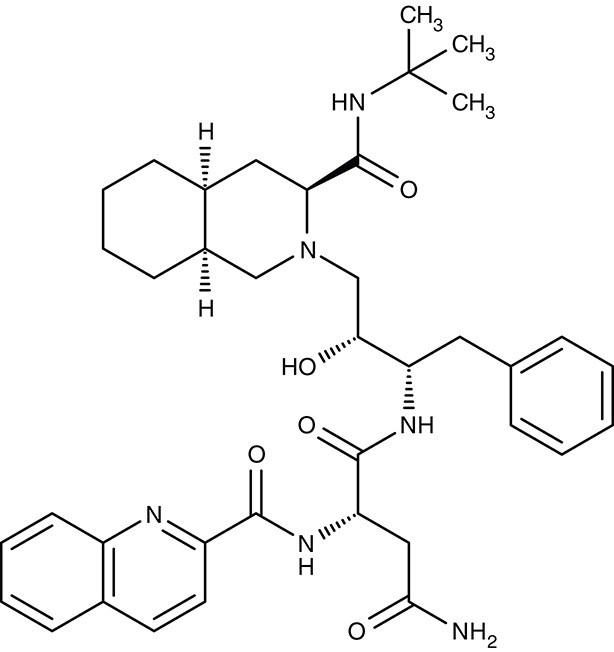 Saquinavir	IC_50_ 13 μM	–	–	–	–	Martin et al., 2012
		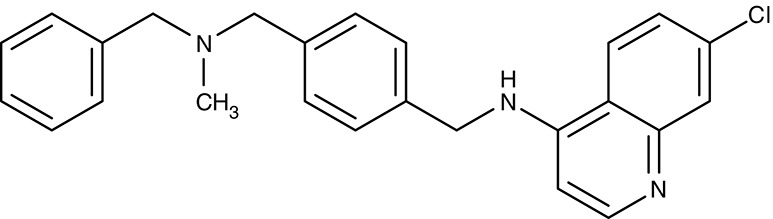 Dibemethin 6a	IC_50_ 69 μM	IC_50_ 26 nM	Trophozoites	–	–	Zishiri et al., 2011
MDR1	*P. falciparum*	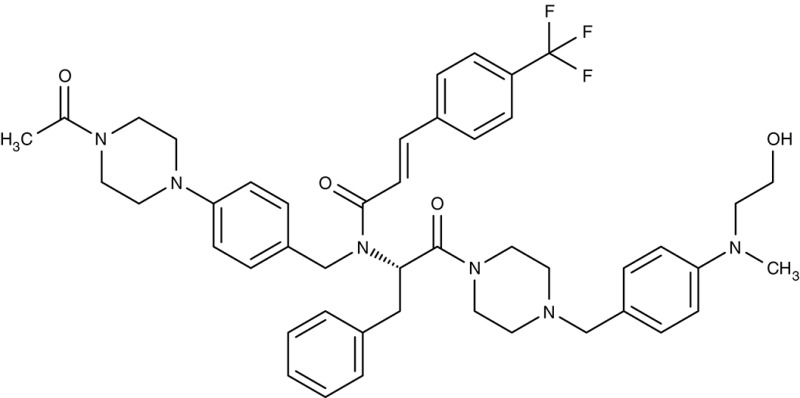 ACT-213615	–	IC_50_ 4 nM	Trophozoites	ED_90_ 8.4 mg kg^−1^ as effective as chloroquine	Mouse	Brunner et al., 2012, 2013
		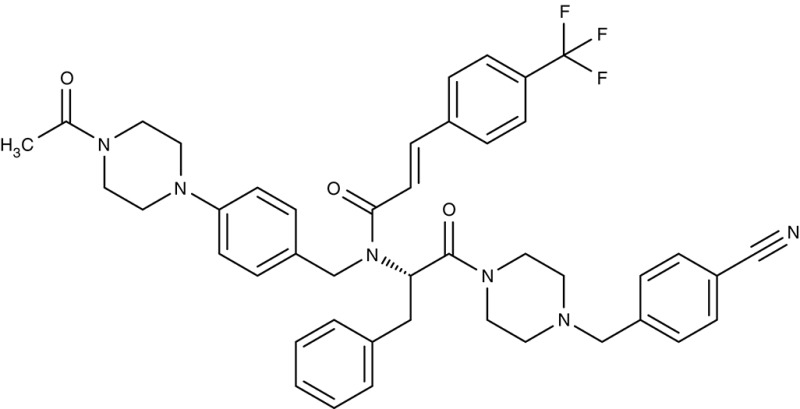 ACT-451840	–	IC_50_ 4 nM	Gametocytes	IC_50_ 2.7 ng ml^−1^ (3.6 nM)	Human	Krause et al., 2016; Le Bihan et al., 2016; Ng et al., 2016

Despite these challenges, all currently used drugs in anti-parasite therapy act at internal sites. It remains to be seen how a shift to *peripheral* or even *indirect* attacks will affect resistance formation.

#### Artemisinins/sarcoplasmic P-type calcium ATPase SERCA/ATP6

Artemisinin (Table 3) and derivatives are the most important antimalarials today. The molecules contain a peroxo moiety. It is discussed that iron^II^ from heme when released from hemoglobin during degradation in the digestive vacuole chemically activates the artemisinins producing reactive oxygen species that damage proteins more or less specifically inside the mitochondria (Asawamahasakda et al., 1994; Moore et al., 2011) or at other sites in the cell including DNA as a target. Evidence further points to a P-type calcium ATPase, SERCA, or ATP6, at the sarcoplasmic endoplasmic reticulum as one of the affected proteins (Eckstein-Ludwig et al., 2003; Naik et al., 2011; Abiodun et al., 2013; Pulcini et al., 2013; Krishna et al., 2014; Nunes et al., 2016). However, there is a mismatch of artemisinin efficiency on the parasites and on heterologously expressed ATP6. In *P. falciparum* cultures, IC_50_-values were 11 and 13 nM tested on a chloroquine resistant (K1) and a sensitive (NF54) strain, respectively (del Pilar Crespo et al., 2008). The artemisinins also showed some potency on several other protozoan parasites, i.e., *T. gondii* (Berens et al., 1998; Jones-Brando et al., 2006; Hencken et al., 2010), *T. brucei, T. cruzi, L. donovani, L. major* (Yang and Liew, 1993; Mishina et al., 2007), and *Babesia gibsoni* (Iguchi et al., 2015) yielding EC_50_-values from 0.36 to 120 μM. For *Leishmania* spp., artemisinin exhibited *in vivo* activity in infected hamster and BALB/c mice models (Ma et al., 2004; Sen et al., 2010; Ghaffarifar et al., 2015). *Naegleria fowleri*, a problematic, cyst-forming amoeba, has been shown to be sensitive to artemisinin *in vitro* (Cooke et al., 1987), whereas treatment failed in a mouse model (Gupta et al., 1995). Purified recombinant *P. falciparum* ATP6, however, could not be directly inhibited by artemisinin or derivatives (Cardi et al., 2010; Arnou et al., 2011), and full inhibition of yeast expressed SERCA of *T. gondii* required high concentrations of 10 μM (Nagamune et al., 2007). The small molecule arterolane (Table 3) has a different scaffold than the artemisinins but equally contains a peroxo group and, thus, should be capable of releasing reactive oxygen species. When tested on *P. falciparum* ATP6 expressed in *Xenopus* oocytes it was found to be clearly less potent than artemisinin with a K_i_-value of 7.7 μM; yet, parasite growth was inhibited a very low IC_50_ of 1.5 nM similar to artemisinin. These mixed results show that ATP6/SERCA is probably not the main target of the artemisinins.

Still, parasite SERCA/ATP6 seems to hold potential as a therapeutic target, because thapsigargin (Table 3), a plant sesquiterpene lactone and general SERCA inhibitor, killed cultured chloroquine resistant and sensitive *P. falciparum* parasites with IC_50_ of 246 and 298 nM (del Pilar Crespo et al., 2008; Abiodun et al., 2013). Thapsigargin further inhibited growth of *T. gondii, Trypanosoma* spp., *L. donovani, E. invadens*, and *Neospora canium* with EC_50_-values in the range of 0.5–39 μM (Kim et al., 2002; Mishina et al., 2007; Martínez-Higuera et al., 2015). Yeast expressed *T. gondii* SERCA was fully inhibited by thapsigargin at 1 μM (Nagamune et al., 2007).

#### Atovaquone/mitochondrial cytochrome bc1 complex

A key function of mitochondria in general is the build-up of a steep proton gradient across the inner mitochondrial membrane, which is used to drive ATP synthesis by the F-type ATPase, or ATP synthase. To this end, the inner membrane harbors a cytochrome bc1 complex. The bc1 proteins use ubiquinone, also called coenzyme Q, as a redox cofactor in electron transfer reactions (Q-cycle), which free four protons that are transported to the intermembrane space in the process (Crofts et al., 1999a,b; Crofts, 2004).

The drug atovaquone (Table 3), a ubiquinone analog and cytochrome bc1 complex inhibitor, is in use against malaria, toxoplasmosis and babesiosis for many years. It interferes with ubiquinone cofactor binding to the Q_0_ site as shown by manifesting resistance mutations in this region of the protein (Fry and Pudney, 1992; Srivastava et al., 1999; McFadden et al., 2000; Kessl et al., 2007; Vallières et al., 2012; Siregar et al., 2015). Combination of atovaquone with proguanil lowers the IC_50_ from 2 nM to 400 pM probably by a synergistic mechanism as proguanil destroys the mitochondrial membrane potential in the presence of an electron transport inhibitor (Srivastava and Vaidya, 1999). Several resistant strains have formed during the use of atovaquone (Hutchinson et al., 1996; Painter et al., 2007; da Cruz et al., 2012). Since the cytochrome bc1 complex is common to mitochondria of all species is was possible to inhibit the growth of other parasites as well. Potency against *T. gondii* was sub-micromolar *in vitro*, i.e., tachyzoites in human foreskin fibroplasts, and *in vivo* using a mouse model with IC_50_-values of 0.14 and 0.85 μM, respectively (Doggett et al., 2012). Similarly, atovaquone acted on *Babesia* spp. *in vitro* and *in vivo* in hamsters (Hughes and Oz, 1995; Wittner et al., 1996; Matsuu et al., 2008). In dog and human studies, combination with the antibiotic azithromycin turned out to be positive (Krause et al., 2000; Birkenheuer et al., 2004; Di Cicco et al., 2012; Checa et al., 2017). Treatment of *L. donovani* infections in a mouse model were less successful resulting only in a 30% lower parasite burden (Croft et al., 1992).

A related hydroxynaphthoquinone compound, buparvaquone (Table 3), is used for the treatment of theileriosis in cattle (McHardy et al., 1985; Minami et al., 1985; Muraguri et al., 1999; Mhadhbi et al., 2010), and horses (Zaugg and Lane, 1989). Anti-leishmanial activity was evaluated *in vitro* for *L. donovani, Leishmania aethiopica, L. major, Leishmania amazonensis, Leishmania mexicana, Leishmania panamensis, L. infantum, Leishmania chagasi, L. braziliensis*, and *L. tropica* promastigotes and amastigotes resulting in IC_50_-values of 0.001–5.495 μM (Mäntylä et al., 2004a,b; Reimão et al., 2012; Jamal et al., 2015). Animal models showed that the *in vivo* efficiency was higher when prodrugs of buparvaquone were applied (Croft et al., 1992; Garnier et al., 2007) or when a nanoliposomal drug preparation was used (da Costa-Silva et al., 2017).

In the search for alternative chemical scaffolds, decoquinate (Table 3), a 4-oxo-quinoline, was tested. However, the overall structure is still similar to ubiquinone. The compound is in use in veterinary medicine against coccidia (Miner and Jensen, 1976; Ricketts and Pfefferkorn, 1993). When tested against blood-stage as well as liver-stage *P. falciparum*, in either case nanomolar IC_50_ values were found at low host cell toxicity (da Cruz et al., 2012). Another potent inhibitor of the cytochrome bc1 complex with good selectivity for *P. falciparum* is the dihydroacridinedione WR249685 (Table 3), which has an IC_50_ of 3 nM on the *in vitro* growth of the parasites (Biagini et al., 2008). Screening of the TCAMS library for cytochrome bc1 complex inhibition yielded one efficient compound, TCMDC-135546 (Table 3), with an IC_50_ of 22 nM on parasite growth (Raphemot et al., 2015). Naphthoquinone esters were derived from the anticancer drug rhinacanthin and showed low nanomolar IC_50_-values on the growth of *P. falciparum*. Notably, the molecules seem to bind to the Q_i_ ubiquinone site of cytochrome bc1 rather than Q_0_ as the above-mentioned compounds (Kongkathip et al., 2010). In a diversity oriented synthesis approach, macrolactame derivatives were generated that yielded nanomolar EC_50_-values and are thought to target the Q_i_ site as well (Comer et al., 2014; Lukens et al., 2015). Finally, more compounds were derived from endochin, an experimental antimalarial of the 1940s, with the aim to improve solubility and metabolic stability in the host. Such compounds were found not to inhibit the human cytochrome bc1 complex but to very efficiently target the Q_i_ site of the cytochrome bc1 complex of *falciparum* and *vivax* plasmodia from various clinical field isolates as well as *T. gondii* and *Babesia microti* (Doggett et al., 2012; Nilsen et al., 2013; Lawres et al., 2016). *Leishmania* spp., however, were much less susceptible to this type of inhibitors with IC_50_-values in the micromolar range (Ortiz et al., 2016).

#### Diamidines/mitochondrial ATP synthase

Similar to the artemisinins in plasmodia, the mode of diamidine action, e.g., pentamidine, in trypanosomes is not fully resolved. Screening of an diamidine library yielded furamidine (Table 3) and related compounds that act comparably to pentamidine against *T. brucei* parasites in mice and rhesus monkeys (Rane et al., 1976; Steck et al., 1982). Further, a prodrug of furamidine, DB289 (Table 3), with better oral availability was developed (Ismail et al., 2003; Ansede et al., 2004). Regarding transporter targeting, it was found that the F1F0-ATPase, i.e., the mitochondrial proton gradient-driven ATP synthase, was inhibited at concentrations around 10 μM and caused a collapse of the mitochondrial membrane potential. The efficiency of diamidines on the growth of trypanosomes, however, is clearly lower, i.e., in the submicromolar range, indicating that ATP synthase is not the main target. It was also suggested that the compounds inhibit other ATPases (Lanteri et al., 2008). DB289 entered phase III clinical trials as the first orally available drug against blood stage human African trypanosomiasis. However, due to manifestation of delayed renal insufficiency in a number of recipients, further development was terminated (Harrill et al., 2012). Two related aza analogs of furamidine (DB820, CPD0801) are still followed up on as they have shown efficiency against *T. brucei* in a mouse model of second stage trypanosomiasis (Wenzler et al., 2009; Ward et al., 2011).

#### G25/mitochondrial choline transport

The notion that a choline-related compound, named G25 (Table 3) carrying two quaternary ammonium moieties spaced by a 16-carbon linker, efficiently kills malaria parasites led to the idea that choline transport might be a valid antimalarial target (Wengelnik et al., 2002; Biagini et al., 2004). Besides *P. falciparum*, also *T. brucei* and *L. mexicana* turned out to be sensitive to G25 (Ibrahim et al., 2011). The mode of action remains unclear. Besides the notion that these compounds inhibit choline transport, it was reported that the mitochondrial structure and function of trypanosomes was affected (Ibrahim et al., 2011; Macêdo et al., 2013). An RNA interference approach in the blood stream form of *T. brucei* hinted at the involvement of a member of a mitochondrial carrier protein family, TbMCP14, which is unrelated to mammalian carriers (Schumann Burkard et al., 2011; de Macêdo et al., 2015).

#### Aminomethylenediphosphonate/vacuolar-type inorganic pyrophosphatase

Vacuolar-type pyrophosphatases, V-PPases, seem to be absent in vertebrates, yet have vital functions in protozoan energy conservation and membrane transport. Accordingly, they may represent suitable drug targets (Rodrigues et al., 2000; Drozdowicz et al., 2003). Yeast-expressed V-PPase from *T. gondii*, TgVP1, was inhibitable by aminomethylenediphosphonate, AMDP (Table 3), with an IC_50_ of 0.9 μM. The presence of V-PPases has also been shown in *P. falciparum* and *T. cruzi* (Urbina et al., 1999; McIntosh et al., 2001). Anti-parasitic, drug-like molecules targeting V-PPases are yet to be found.

### Targeting drug efflux transporters

Drug resistance due to expedited export of the compounds is a key factor in pharmacotherapy not only of anti-infectives but in general. The loss of drug action may be reversed by inhibition of the responsible efflux transporter.

#### Verapamil, (dimeric) quinine, saquinavir/digestive vacuole chloroquine resistance transporter

The discovery of chloroquine was a breakthrough in malaria therapy. It acts by inhibiting heme detoxification in the form of polymerized hemozoin in the digestive vacuole (Foley and Tilley, 1997). Promoted by monotherapeutic use and widespread underdosing in eradication programs, however, resistant *P. falciparum* strains were selected over the years rendering chloroquine largely useless today (Wellems et al., 1991; Waller et al., 2003). Resistance mutations were found in one particular gene of unknown function at that time. It turned out that the mutations resulted in a gain-of-function transporter shuttling chloroquine out of the digestive vacuole at a strongly increased rate (Fidock et al., 2000; Lakshmanan et al., 2005). The physiological role of the chloroquine-resistance-transporter, CRT, is still elusive. However, a variety of amino acids, polyamines and peptides have been found to be CRT substrates (Juge et al., 2015). Several attempts have been undertaken to block CRT with the aim to reverse chloroquine resistance. The calcium channel blocker verapamil (Table 3) was shown to inhibit CRT expressed in *Xenopus* oocytes with an IC_50_ of 30 μM (Ye and van Dyke, 1988, 1994; Tanabe et al., 1989). In the same system, quinine, a natural product and chloroquine analog, yielded an IC_50_ of 48 μM (Martin et al., 2009), and the antiretroviral drug saquinavir was effective at 13 μM (Martin et al., 2012). Chemical synthesis of dimeric quinines lowered the IC_50_ to 1 μM on CRT-expressing oocytes and efficiently inhibited parasite growth in the nanomolar range (Hrycyna et al., 2014).

The resistance reversing effect of verapamil cannot be exploited in humans, because the required dose would be too high to be tolerable (Ye and van Dyke, 1988). One approach was to develop a drug-like compound with dual functionality, i.e., blockade of hemozoin formation plus inhibition of CRT (Burgess et al., 2006; Kelly et al., 2009). The obtained dibemethin derivates (Table 3) showed IC_50_ values of 26 nM on parasite growth, yet potency on the isolated CRT protein was only 69 μM. Bioavailability appeared sufficient after oral administration in mice (Zishiri et al., 2011).

#### ACT-213615, ACT-451840/digestive vacuole multidrug resistance transporter 1

Based on similarity to human multidrug resistance transporters in terms of sequence and function another transporter of the digestive vacuole of malaria parasites was termed multidrug resistance transporter 1, MDR1 (Foote et al., 1989; Cowman et al., 1991). MDR1 transport is directed into the digestive vacuole. This way, mefloquine, artemisinin, and artesunate are thought to be trapped by compartmentalization preventing the drugs from hitting their target in the sarcoplasmic endoplasmic reticulum (Reed et al., 2000; Pickard et al., 2003; Price et al., 2004; Rohrbach et al., 2006). Resistance was increased in strains with multiple copies of the *pfmdr1* gene (Cowman et al., 1994; Pickard et al., 2003). For quinine in turn, it is required that MDR1 is functional to deliver the compound to its site of action inside the digestive vacuole. Therefore, a transport decreasing N1042D mutation of MDR1 was made responsible for quinine resistance (Rohrbach et al., 2006). Apart from transport of antimalarial drugs, MDR1 appears to be vital for the malaria parasite, because in cell viability screening and subsequent analyses, two small-molecule compounds were found to target MDR1. ACT-213615 (Table 3) is proposed to inhibit either PfMDR1 directly or a regulating protein upstream of it yielding an EC_50_ value of 4 nM on parasite growth (Brunner et al., 2012, 2013). The second, highly related compound, ACT-451840 (Table 3), was even more potent and a single nucleotide polymorphism in the MDR1 gene was identified that turned out to be responsible for resistance against the compound. The compound was well tolerated in first clinical trials, however, none of the eight tested subjects could be completely freed from parasites (Krause et al., 2016; Le Bihan et al., 2016; Ng et al., 2016). The physiological substrates and function of MDR1 remain to be established.

## Conclusion

The identification of anti-parasitic targets at the parasite's plasma membrane or even at host membranes opens up options for *peripheral* or *indirect* therapeutic attacks (Figure 1). This approach holds the potential to limit resistance formation because the only remaining line of defense is mutational modification of the drug binding site at the target protein. It is thinkable that massive upregulation of target protein expression may also reduce efficiency, yet at high energetic cost for the parasite.

Still, most currently addressed targets are located inside the parasite. Here, specificity must be taken into account. Drugs affecting vital functions by hitting multiple targets, e.g., the artemisinins by eliciting oxidative stress, are hard to defend by the parasite at the various target sites. However, several ways exist to interfere with drug action by acting directly on the molecular entity. Drug uptake can be prevented if transporters are required to deliver the compound to the cytosol, e.g., pentamidine via TbAQP2. The drug can be chemically modified or compartmentalized, e.g., artesunate transport into the digestive vacuole by MDR1. Finally, the compound can be shuttled out to keep the concentration below a harmful level, e.g., chloroquine via CRT. Drug metabolism and transport issues cannot be fully appreciated beforehand. Hence, in the search for potent anti-infectives against parasites and bacteria alike, phenotypic screenings have proven more successful than specific approaches in designing inhibitors for a selected target protein.

The ideal anti-parasitic drug compound should i. reach the site of action independent of parasite transport proteins, ii. be specific for the parasite but not necessarily for a single target protein, iii. act fast, iv. be metabolically stable, and v. be safe for the patient. Meeting such demands and circumventing the influence by the parasite may be eased by addressing parasite-specific proteins at the periphery.

## Author contributions

AM, HE, and EB jointly wrote the manuscript and prepared the figures.

### Conflict of interest statement

The authors declare that the research was conducted in the absence of any commercial or financial relationships that could be construed as a potential conflict of interest.
